# Synthesis and *In Vitro* Biological Evaluation of 1,3,4-Oxadiazol-2(3*H*)-one and Tetrahydropyridazine-3,6-dione Derivatives of Fatty Acids

**DOI:** 10.3797/scipharm.1503-10

**Published:** 2015-06-09

**Authors:** Mohammad F. Hassan, Abdul Rauf, Asif Sherwani, Mohammad Owais

**Affiliations:** 1 Department of Chemistry, Aligarh Muslim University, Aligarh 202002, Uttar Pradesh, India; 2 Interdisciplinary Biotechnology Unit, Aligarh Muslim University, Aligarh 202 002, India

**Keywords:** 1,3,4-Oxadiazol-2-one, Tetrahydropyridazine-3,6-dione, Fatty acids, Cytotoxicity, SAR

## Abstract

Herein we report saturated and unsaturated fatty acid derivatives of 1,3,4-oxadiazol-2(3*H*)-one and tetrahydropyridazine-3,6-dione as new potential anticancer agents. All the synthesised compounds were characterised by IR, ^1^H-NMR, ^13^C-NMR, and mass spectral data. The relative sensitivity of three cancer cell lines varied depending on the nature of the compound. Among the most effective anticancer compounds studied, **3b** and **6b** displayed remarkable anticancer activity against the MDA-MB-231 and KCL-22 lines, respectively. On the other hand, compound **3c** was found to be most sensitive to nearly all the tested cell lines, MDA-MB-231, KCL-22, and HeLa.

## Introduction

**Fig. 1:**
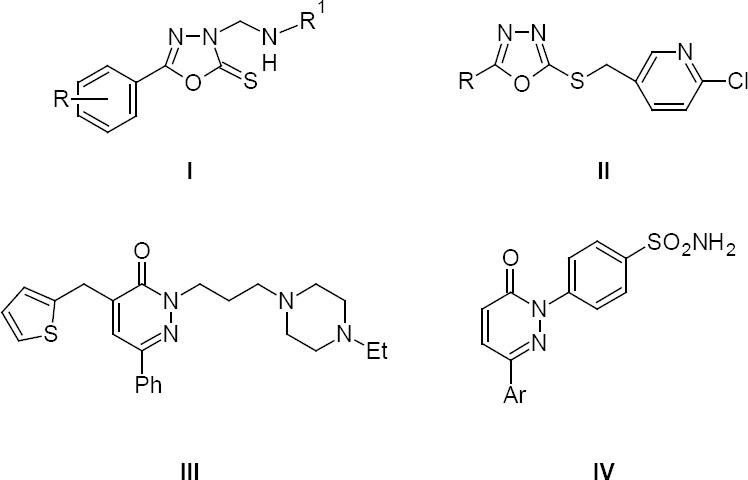
Previously reported compounds that are structurally related to the target compounds

For many years, cancer has been one of the major causes of death. According to the International Agency for Research on Cancer (IARC), an estimated 14.1 million new cancer cases and 8.2 million cancer-related deaths occurred in 2012 and cancer will alone give rise to an estimated 21.1 million incident cases and 13.2 million deaths by 2030 [1]. Today, the discovery and development of new anticancer drugs is the focal point and research in the field of development of potent, selective, and less toxic anticancer drugs has been increased [2]. Compounds containing a 1,3,4-oxadiazole ring system have also attracted a lot of attention due to their wide range of biological activity [3–8]. Some derivatives of the 1,3,4-oxadiazole scaffold have been reported to exhibit remarkable anticancer activities [9, 10]. Recently, a series of 1,3,4-oxadiazole derivatives (**I**) have been reported by the Shahzad group as thymidine phosphorylase inhibitors (TPI) which have the ability to suppress the formation of new blood vessels and stop tumor growth [11]. Zheng *et al*. reported 2-chloropyridine derivatives bearing a 1,3,4-oxadiazole moiety (**II**), which showed remarkable antiproliferative activity [12]. Among the six-membered heterocyclic compounds, pyridazinone derivatives are recognised as a versatile scaffold with a wide range of pharmaceutical activities [13–19]. Literature studies revealed that substituted pyridazinone derivatives possessed anticancer activity against various cell lines [20–22]. More recently, certain pyridazine-3(2*H*)-one derivatives such as (**III**) and (**IV**) (Fig. 1) were found to be promising anticancer agents [23, 24]. Besides, a number of modified fatty acid analogues play a unique role in the prevention of cancer and have been a potential treatment of cancer [25–27]. Understanding these facts and as part of our efforts to develop pharmacologically important fatty acid derivatives [3, 28, 29], we try to combine the 1,3,4-oxadiazol-2(3*H*)-one and tetrahyropyridazine-3,6-dione moieties with long-chain fatty acids by a simple one-pot reaction. Easy absorbance of fatty acids by the human body will lead to more intake of heterocycles associated with fatty acids and further, the long chain fatty acids increase the accumulation and retention of drugs by increasing the affinity and permeability of the cell membranes as reported in the case of other compounds [27]. Our strategy was based upon the formation of a heterocyclic moiety at the fatty acid chain by synthesizing a reactive hydrazide moiety followed by condensation using 1,1’-carbonyldiimidazole (CDI) (Scheme 1) and succinyl chloride (Scheme 2) under very mild conditions. These new compounds, **3a**–**d** and **6a**–**c**, were evaluated for *in vitro* anticancer activity against MDA-MB-231 (Breast), KCL-22 (Lymphoblastoid), and HeLa (cervical) cell lines using the MTT assay.

**Sch. 1:**
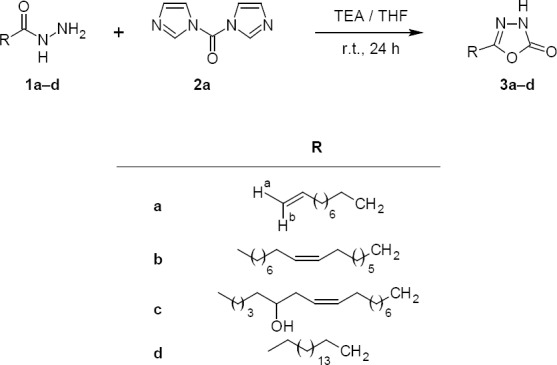
Synthetic route for the target compounds **3a–d**

**Sch. 2:**
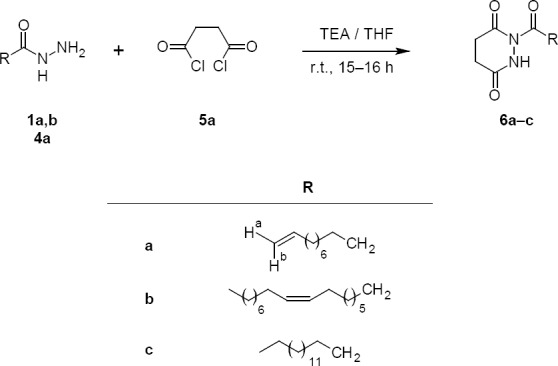
Synthetic route for the target compounds **6a–c**

## Results and Discussion

### Chemistry

The synthetic route for the preparation of new fatty acid derivatives of 1,3,4-oxadiazol-2(3*H*)-one is outlined in Scheme 1.

The target compounds **3a–d** were synthesized by the one-pot cyclisation of the fatty acid hydrazide under mild conditions. The starting compounds, **1a–d**, were prepared by refluxing the methyl esters of the fatty acid with hydrazine hydrate in methanol [30]. The compounds **3a–d** were synthesized by addition of CDI (**2a**) into the stirred THF solution of **1a–d** in the presence of triethylamine (TEA) at room temperature under dry conditions. The structure of all the synthesized compounds **3a–d** were confirmed by FTIR, ^1^H-NMR, ^13^C-NMR, and mass spectra. The IR spectrum of compound **3a** showed a broad absorption band at 3260 cm^-1^ which is characteristic of the N-H group and absorption bands at 2927 cm^-1^ and 2856 cm^-1^ were the unsymmetrical and symmetrical stretching bands of C-H of the fatty acid chain, respectively. The bands at 1781 cm^-1^ and 1634 cm^-1^ corresponded to the carbonyl (CO) and C=N groups, respectively. In the ^1^H-NMR study of **3a**, the disappearance of the doublet ^1^H-NMR peak of the free -NH_2_ of the fatty acid hydrazide at δ 4.13 and the appearance of a broad singlet at δ 9.15 confirmed the presence of the NH proton of 1,3,4-oxadiazol-2(3*H*)-one system. Further, three olefinic protons like (H_a_, H_b_, and H) were observed at δ 4.92, 4.99, and 5.80 respectively. The ^13^C-NMR spectra showed two peaks at 139.1 and 114.2 which corresponded to the olefinic carbons (C_9_-C_10_) of the alkyl chain, respectively. Besides, peaks at 158.4 and 155.6 were recorded for two-ring carbons of 1,3,4-oxadiazol-2(3*H*)-one ring carbons which further confirm the structure. A series of compounds **6a–c** were synthesized by dropping succinyl chloride (**5a**) solution of dichloromethane (DCM) into the stirred DCM solution of **1a–b**, **4a**, and TEA at room temperature (Scheme 2). The FTIR spectra of compound **6a** showed an absorption band at 3216 cm^-1^ which was characteristic of the –NH proton, and absorption bands at 1667 cm^-1^ and 1732 cm^-1^ were characteristic of the cyclic amide carbonyl (NHCO) and CO groups. The bands at 2924 cm^-1^ and 2858 cm^-1^ were the C-H stretching frequencies of the alkyl chain. The ^1^H-NMR spectrum was more informative for structural determination. Diagnostic peaks of two multiplets of compound **6a** at δ 2.69–2.58 were assigned to two methylene moieties of the ring while a singlet at δ 8.81 confirmed the (NHCO) proton. In the ^13^C-NMR spectra, two peaks observed at 173 and 170.23 were the characteristic peaks of two carbonyl groups of the tetrahydropyridazine-3,6-dione ring, whereas the peaks at 168.6, 139.1, and 114.1 were observed for the carbonyl carbon and olefinic carbons (C_10_-C_11_) of the fatty acid alkyl chain, respectively. Similarly, the structures of other compounds were confirmed by their spectral data.

### In Vitro Cytotoxicity Screening

**Fig. 2:**
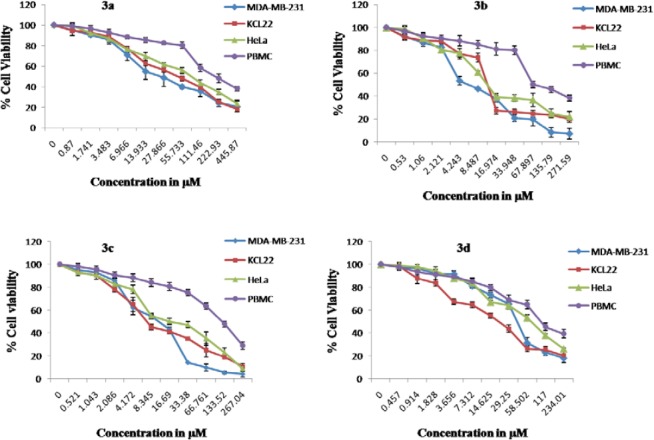
Dose-dependent effects of **3a–d** on the cell viability of MDA-MB-231, KCL-22, HeLa, and PBMC cell lines. Data shown are mean ± standard error of at least four independent experiments

**Fig. 3:**
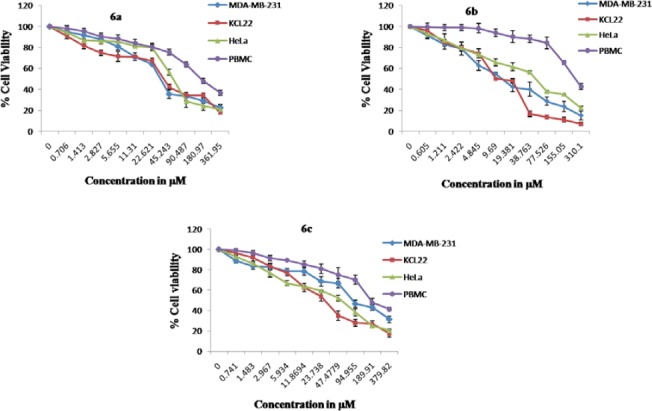
Dose-dependent effects of **6a–c** on the cell viability of MDA-MB-231, KCL-22, HeLa, and PBMC cell lines. Data shown are mean ± standard error of at least four independent experiments

**Fig. 4:**
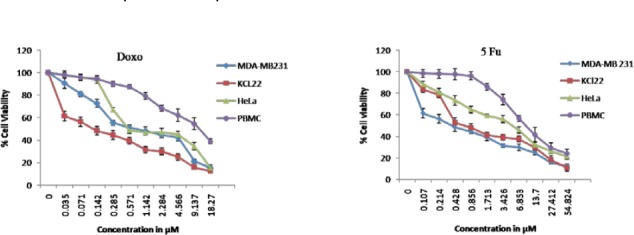
Dose-dependent effects of Doxorubicin and 5-Fu on the cell viability of MDA-MB-231, KCL-22, HeLa, and PBMC cell lines. Data shown are mean ± standard error of at least four independent experiments

The newly synthesized compounds, **3a–d** and **6a–c**, combined the benefits of the aforementioned 1,3,4-oxadiazol-2(3*H*)-one and tetrahydropyridazine-3,6-dione moieties with their fatty acid substituent to give a new scaffold. To check the anticancer potency, the compounds **3a–d** and **6a–c** were screened *in vitro* for cytotoxicity against MDA-MB-231 (Breast), KCL-22 (Lymphoblastoid), and HeLa (cervical) cell lines, while PBMC (peripheral blood mononuclear cells) were used as a normal cell. Doxorubicin and 5-Fluorouracil were used as standard cytotoxic drugs [31]. A period of 48 hours of drug exposure was chosen to test cytotoxicity. The MTT cell viability assay based on the conversion of the soluble yellowish MTT to the insoluble purple formazan crystal by active mitochondrial lactate dehydrogenase of living cells was used for the measurement of cell proliferation. The inhibitory concentration (IC_50_) of **3a–d** and **6a–c** compounds were screened against the MDA-MB-231, KCL-22, and HeLa cell lines as given in Table 1. The graphs representing the dose-dependent effects of compounds **3a–d** and **6a–c** are shown in Fig. 2 and Fig. 3. The effect of standard drugs (Doxorubicin and 5-Fluorouracil) on the MDA-MB-231, KCL-22, and HeLa cell lines, and PBMC are represented in Fig. 4. The data given in (Table 1) reveals that all newly screened compounds reduced the MDA-MB-231, KCL-22, and HeLa cell lines up to 50% at different IC_50_ doses. Further, all the synthesized compounds **3a–d** and **6a–c**, when tested with normal cells (PBMC) were found to be non-cytotoxic (IC_50_ < 50 µM).

**Tab. 1:**
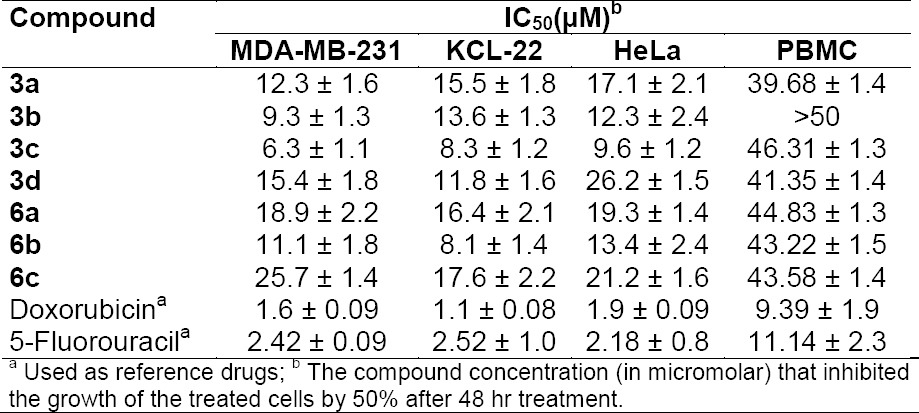
*In vitro* anticancer activities (IC_50_ µM) of compounds **3a–d** and **6a–c** against MDA-MB-231, KCL-22, HeLa, and PBMC cell lines

### Structure-Activity Relationship (SAR)

**Fig. 5:**
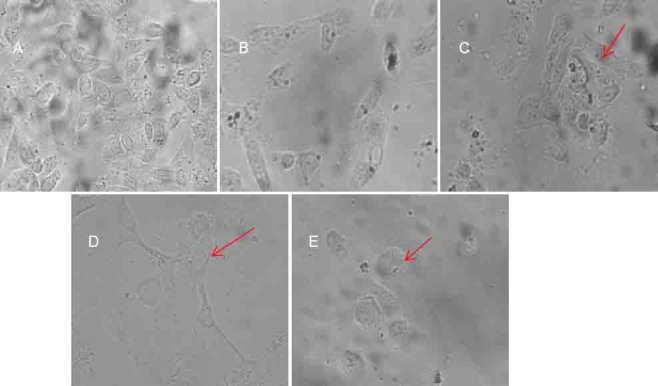
Fluorescence microscope images of MDA-MB-231 cells after 48 hours of incubation with 10 mg mL^-1^ of compounds (B) **3d**, (C) **3a**, (D) **3b**, (E) **3c**. (A) Untreated control. The arrow shows cells which are going to die or, apoptotic cells

**Fig. 6:**
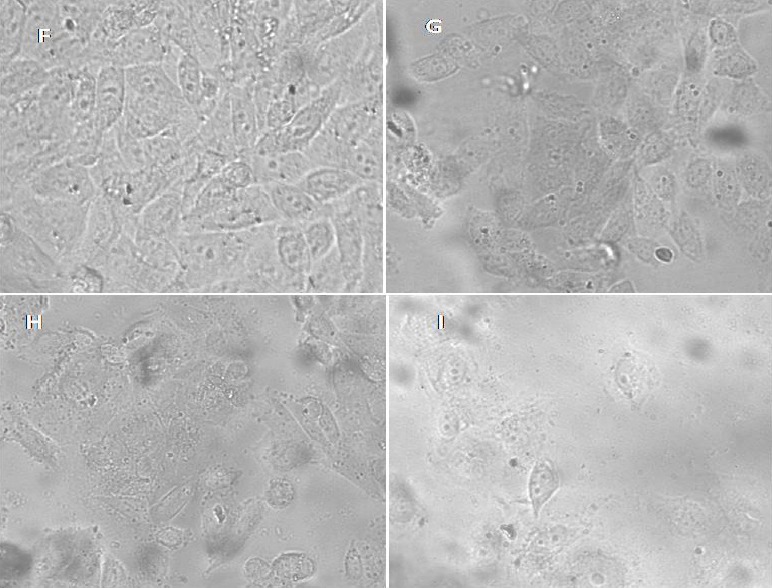
Fluorescence microscope images of MDA-MB-231 cells after 48 hours of incubation with 10 mg mL^-1^ of compounds (G) **6c**, (H) **6a**, (I) **6b**. (F) Untreated control

The relationship between cytotoxic activity and the compounds was observed (see Table 1). From the IC_50_ values, all the tested compounds showed well-to-moderate activities on all the tested cell lines in the range from 6.3 to 26.2 µM. Our result shows that the nature of the fatty acid chain plays an important role in determining the anticancer activity of drugs. When we changed the fatty acid from saturated octadecanoic acid (stearic acid) or hexadecanoic acid (palmitic acid) to unsaturated undec-10-enoic acid and (9*Z*)-octadec-9-enoic acid (oleic acid), there was an increase in anticancer activity (**3d** vs. **3a–c** and **6c** vs. **6a,b**) against nearly all three tested cell lines, except in the case of the KCL-22 cell line in which the saturated analogues **3a** and **3b** were found to be more cytotoxic (Table 1). The position of the (C=C) double bond also determines the cytotoxic behaviour of the compounds. The compounds **3b**, **3c**, and **6b** containing a *cis*-double (C=C) bond at C-8 have a remarkable inhibitory effect compared to compounds **3a** and **6a** with a terminal (C=C) bond at C-9 and C-10 positions, respectively. Further, the presence of a polar hydroxyl group remarkably increases the cytotoxicity of compounds in comparison to compounds with a non-hydroxy fatty acid chain. For example, among the two unsaturated compounds **3b** and **3c**, **3c** exhibited more potent anticancer activity (IC_50_= 6.3, 8.3, 9.6) than **3b** (IC_50_= 9.3, 13.6, 12.3) against three cell lines. It is more probable that the presence of the hydroxyl group in **3c** is responsible for this enhanced activity. The fluorescence microscopy micrographs of MDA-MB-231 cell lines were also in good agreement with the result of the IC_50_ table. The fluorescence microscopy micrographs (Fig. 5 and Fig. 6) showed the apoptosis induced by compounds **3a–d** and **6a–c.** The results of the fluorescence microscopy micrographs showed that among the compounds **3a–d** and **6a–c**, the cytotoxic potency follows the following order– **3c** > **3b** > **3a** > **3d** and **6b** > **6a** > **6c**. In short, the presence of the toxophoric –N=C-O- linkage in the 1,3,4-oxadiazole nucleus and cyclic amide –NH-C=O- of the tetrahydropyridazine-3,6-dione moiety may be the key factor for determining the cytotoxic activity. The enhanced cytotoxicity could be attributed to the presence of long fatty acid chains which increase the accumulation and retention of drugs by increasing the affinity and permeability for cancer cell membranes. The unsaturation in the fatty acid chain, and particularly the hydroxyl group, led to compounds with enhanced cytotoxic activity.

### Cytotoxicity Assay

#### Maintenance of Cells

The MDA-MB-231, KCL-22, and HeLa cell lines were maintained in RPMI 1640 (Sigma Aldrich, USA) culture medium supplemented with 10% heat-inactivated fetal calf serum (Sigma Aldrich, USA), and antibiotic antimycotic solution (Sigma Aldrich, USA).

#### Blood Peripheral Mononuclear Cell Isolation

Fresh blood (20–15 mL) was provided by the Blood Bank JNMC AMU, Aligarh. After dilution with the same volume of PBS, the diluted blood sample was layered on the Ficoll-Histopaque (Sigma Aldrich, USA). The mixture was centrifuged at 400×*g* for 30 min at 20–22°C followed by separation of the lymphocyte layer. Then the lymphocyte layer was washed and pelleted down with three volumes of PBS twice and resuspended with RPMI-1640 media with antibiotic and antimycotic solution 10%, v/v fetal calf serum (FCS).

#### MTT Assay

The cells were plated at a density of (5×10^4^ / well) in 96-well plates and cultured for 24 hours at 37°C. The cytotoxicity of compounds was determined by the MTT assay which involves the percentage viability of a cell in response to variable doses of compounds. After incubation for 48 hours, cell proliferation was measured by adding 20 μL of MTT dye (5 mg/mL in phosphate-buffered saline) per well. The plates were incubated for 4 hours more at 37°C in a humidified chamber containing 5% CO_2_. Active mitochondrial dehydrogenases of living cells reduced the yellow tetrazolium salt (dye) to an insoluble purple formazan crystal. Formazan crystals formed due to the reduction of tetrazoline dye by viable cells in each well which were dissolved in 150 μL dimethyl sulfoxide, and absorbance was read at 570 nm using a plate reader (Bio-Rad). The percentage of inhibition of cell viability was determined with reference to the untreated control. To determine the IC_50_ value, a linear regression analysis method was used.

## Experimental

### Chemical and Instruments

Undec-10-enoic and (9*Z*)-octadec-9-enoic acids were purchased from Fluka Chemicals (Buck, Switzerland). (12*R*,9*Z*)-12-hydroxyoctadec-9-enoic (Ricinoleic, 98%) acid was isolated from *Ricinus communis* seed oil by Gunston’s (1954) partition procedure [32]. 1,1’-carbonyldiimidazole (CDI) and triethylamine (TEA) were purchased from Sigma-Aldrich (India). The solvents like tetrahydrofurane (THF) and dichloromethane (DCM) were dry and AR grade and were purchased from S D Fine-Chem. Ltd. (India). Anhydrous sodium sulphate (Na_2_SO_4_) was used as a drying agent. The progress of reactions was monitored by TLC (thin layer chromatography). TLC was done on glass plates (20 × 5 cm) with a layer of Silica Gel G (Merck, Mumbai, India, 0.5-mm thickness). The spots of the compounds were observed upon exposure to iodine vapour. A mixture of petroleum ether–ethyl acetate–acetic acid (70:30:1, v/v) was used as developing solvent. Column chromatography was carried out on silica gel (Merck, Mumbai, India, 60–120 mesh). The melting points of the synthesized compounds were determined by the open tube capillary method and are uncorrected. The ^1^H-NMR and ^13^C-NMR spectra of the compounds were recorded on a Bruker AVANCE II 400 NMR Spectrometer. The ^1^H-NMR spectra of the compounds were recorded at 400 MHz in CDCl_3_ using TMS as internal standard and THE ^13^C-NMR spectrum was recorded at 100 MHz in CDCl_3_ with CDCl_3_ (δ=77.00), and chemical shifts (δ) are quoted in ppm. The mass spectra were obtained on an LC-MS spectrometer Model Q-TOF Micro Waters. FTIR (Fourier transform infrared) spectra were recorded on the Perkin Elmer FTIR Spectrometer. Fluorescence images were obtained on a fluorescence microscope (Axio, HBU 50/AC; Zeiss, Gottingen, Germany).

### Synthesis Procedure for Fatty Acid Hydrazides (1a–d, 4a)

The fatty acid hydrazides **1a–d** and **4a** were synthesized in a similar manner to the literature [30].

### Synthesis Procedure of 5-Substituted-1,3,4-oxadiazol-2-one Derivatives (3a–d)

To a 50 ml dry THF solution of fatty acid hydrazides **1a**–**d** (5 mmol) was added (5 mmol) of triethylamine (TEA) followed by (7 mmol) of **2a** (CDI) in one portion. The resulting mixture was stirred 24 hours at room temperature (Scheme 1). The progress of the reaction was monitored by TLC. The volatiles were then removed in a vacuum and the residue was dissolved in diethyl ether. The organic layer was washed with aqueous 1 M HCl (2×50 ml), saturated aqueous NaHCO_3_ (2×50 ml), and saturated NaCl (2×50 ml) solutions and dried over anhydrous Na_2_SO_4_. The isolated oily crude products **3a–d** were obtained in 55 to 65% yields and were purified by column chromatography using the mixture of *n*-hexane–ethyl acetate (93:7, V/V) as eluent. All the compounds were characterized by their spectral data.

#### 5-(Dec-9-en-1-yl)-1,3,4-oxadiazol-2(3H)-one (3a)

Yellow oily liquid, yield = 60%, IR (KBr, cm^-1^): 3260 (N-H), 2927 (C-H asymm.), 2856 (C-H symm.), 1781 (C=O), 1634 (C=N). ^1^H-NMR (400 MHz, CDCl_3_): δ 9.15 (s, 1H, NH), 5.80 (tdd, 1H, JH^_8^CH_2_= 6.6 Hz, J_H-Hb_ = 10.2 Hz, J_H-Ha_ = 17.1 Hz, CH_2_=C*H*-), 4.99 (dd, 1H, J_Hb-H_ = 10.2 Hz, J_Hb-Ha_ = 2.6 Hz, *H_b_*C=CH-), 4.92 (dd, 1H, J_Ha-H_ = 17.1 Hz, J_Ha–Hb_ = 2.6 Hz, *H_a_*C=CH-), 2.54 (t, 2H, J = 7.0 Hz, C*H_2_*-CO), 2.0 (m, 2H, C*H_2_*-CH=CH_2_), 1.68 (m, 2H, C*H_2_*-CH_2_-CO), 1.34 (br.s, 10H, chain-C*H_2_*). ^13^C-NMR (CDCl_3_, Proton decoupled): δ 158.4, 155.6 (2C, ring), 139.1 (C_9_, H*C*=CH_2_), 114.2 (C_10_, HC=*C*H_2_), 33.7 (C_8_, H_2_*C*-CH=CH_2_), 29.7, 29.2, 29.0, 28.8, 28.7, 26.3, 25.3 (C_1_-C_7_, chain-*C*H_2_). ESI/MS (*m/z)*: 224.2 (M^+^).

#### 5-[(8Z)-Heptadec-8-en-1-yl]-1,3,4-oxadiazol-2(3H)-one (3b)

Yellow oily liquid, yield = 55%, IR (KBr, cm^-1^): 3258 (N-H), 2927 (C-H asymm), 2853 (C-H symm.), 1784 (C=O), 1635 (C=N). ^1^H-NMR (400MHz, CDCl_3_): δ 8.52 (s, 1H, NH), 5.34 (m, 2H, -C*H*=C*H*-), 2.24 (t, 2H, J = 7.5 Hz, C*H_2_*-CO), 2.0 (m, 4H, -C*H_2_*-CH=CH-C*H_2_*-), 1.64 (m, 2H, C*H_2_*-CH_2_-CO), 1.25 (br.s, 20H, chain-C*H_2_*), 0.88 (dist.t, 3H, terminal-C*H_3_*). ^13^C-NMR (CDCl_3_, Proton decoupled): δ 158.3, 154.6 (2C, ring), 130.0 (C_9_,-*C*H=CH-), 129.7 (C_10_,-CH=*C*H-), 33.8, 32.1, 30.3, 30.1, 29.8, 29.6, 29.2, 28.8, “two signals hidden“, 27.3, 24.7, 23.0, 22.7 (C_2_-C_8_, C_11_-C_17_, chain-*C*H_2_), 14.2 (C_17_, *C*H_3_). ESI/MS (*m/z)*: 322.4 (M^+^).

#### 5-[(8Z,11R)-11-Hydroxyheptadec-8-en-1-yl]-1,3,4-oxadiazol-2(3H)-one (3c)

Yellow oily liquid, yield = 55%, IR (KBr, cm^-1^): 3456 (O-H), 3263 (N-H), 2926 (C-H asymm.), 2859 (C-H symm.), 1784 (C=O), 1640 (C=N). ^1^H-NMR (400MHz, CDCl_3_): δ 9.1 (s, 1H, NH), 5.28 (m, 2H, -C*H*=C*H*-), 3.52 (m, 1H, C*H*-OH), 2.37 (t, 2H, J = 7.4 Hz, C*H_2_*-CO), 2.23 (s,1H,O*H*), 2.10 (m, 4H, -C*H_2_*-CH=CH-C*H_2_*-), 1.72 (m, 2H, C*H_2_*-CH_2_-CO), 1.29 (br.s, 18H, chain-C*H_2_*), 0.83 (dist.t, 6H, terminal-C*H_3_*). ^13^C-NMR (CDCl_3_, Proton decoupled): δ 158.4, 155.5 (2C, ring), 133.1 (C_8_, -*C*H=CH-), 125.6 (C_9_, -CH=*C*H-), 71.2 (C_11_, *C*HOH), 37.0, 35.4, 31.8, 31.6, 30.2, 29.6, 29.5, “one signal hidden“, 29.1, 28.9, 28.7, 23.6, 21.9 (C_1_-C_7_, C_10_, C_12_-C_16_, chain-*C*H_2_), 14.0 (C_17_, *C*H_3_). ESI/MS (*m/z)*: 338.5 (M^+^).

#### 5-Heptadecyl-1,3,4-oxadiazol-2(3H)-one (3d)

Yellow oily liquid, yield = 65%, IR (KBr, cm^-1^): 3256 (N-H), 2925 (C-H asymm.), 2853 (C-H symm.), 1778 (C=O), 1637 (C=N). ^1^H-NMR (400MHz, CDCl3): δ 8.84 (s, 1H, NH), 2.38 (t, 2H, J = 7.5 Hz, C*H_2_*-CO), 1.70 (m, 2H, C*H_2_*-CH_2_-CO), 1.32 (br.s, 28H, chain-C*H_2_*), 0.90 (dist.t, 3H, terminal-C*H_3_*). ^13^C-NMR (CDCl_3_, Proton decoupled): δ 159.6, 155.4 (2C, ring), 32.7, 32.2, 31.1, 30.2, 29.6, 29.6, 29.4, 29.2, 28.8, 28.6, 27.9, 27.3, 27.1, 26.6, 25.2, 22.7 (C_1_-C_16_, chain-*C*H_2_), 13.9 (C_17_, *C*H_3_). ESI/MS (*m/z)*: 323.8 (M^+^).

## Synthesis Procedure of 1-Substituted-tetrahydropyridazine-3,6-dione Derivatives (6a–c)

A typical process involves the dropping **5a** (5 mmol) solution of dry dichloromethane (DCM) (10 ml) into the stirred solution of fatty acid hydrazides **1a**–**b** or **4a** (5 mmol) and TEA (5 mmol) in dry DCM (50 ml). The reaction mixture was stirred overnight at room temperature maintaining the slow dropping of **5a** (Scheme 2). After completion of the reaction (as monitored by TLC), the solvent was evaporated under reduced pressure and the residue was dissolved in DCM. The organic layer was washed with water (3×100 ml) and dried over anhydrous sodium sulphate. The solid crude products were obtained in good yields (68 to 70%) after evaporation of the organic solvent. The isolated compounds **6a–c** were purified by column chromatography using the mixture of n-hexane-ethyl acetate (90:10, v/v) as eluent. All the pure compounds were characterized by their spectral analysis.

### 1-(Undec-10-enoyl)tetrahydropyridazine-3,6-dione (6a)

White powder, yield = 70%, m.p. = 68–70°C, IR (KBr, cm^-1^): 3216 (N-H), 2924 (C-H asymm.), 2858 (C-H symm.), 1732 (C=O), 1667 (CONH, lactam ring). ^1^H-NMR (400 MHz, CDCl_3_): δ 8.81 (s, 1H, NH), 5.80 (tdd, 1H, JH^_ 9^CH_2_= 6.8 Hz, J_H-Hb_ = 10.2 Hz, J_H-Ha_ = 17.0 Hz, CH_2_=C*H*-), 5.01 (dd, 1H, J_Hb-H_ = 10.2 Hz, J_Hb-Ha_ = 3.4 Hz, *H_b_*C=CH-), 4.93 (dd, 1H, J_Ha-H_ = 17.0 Hz, J_Ha–Hb_ = 3.4 Hz, *H_a_*C=CH-), 2.69-2.58 (m, 4H, -C*H_2_*-C*H_2_*-, ring), 2.24 (t, 2H, J = 7.4 Hz, C*H_2_*-CO), 2.0 (m, 2H, C*H_2_*-CH=CH_2_), 1.63 (m, 2H, C*H_2_*-CH_2_-CO), 1.31 (br.s, 10H, chain-C*H_2_*). ^13^C-NMR (CDCl_3_, Proton decoupled): δ 173.0, 170.2 (2*C*=O, ring), 168.6 (*C*=O, chain), 139.1 (C_10_, H*C*=CH_2_), 114.1 (C_11_, HC=*C*H_2_), 34.0 (C_2_), 33.7, 31.8 (2C, -*C*H_2_-*C*H_2_-, ring), 29.5, 29.4, 29.2, 29.0, 28.9, 28.5, 25.3 (C_3_-C_9_, chain-*C*H_2_). ESI/MS (*m/z)*: 281.5 (M^+^).

### 1-[(9Z)-Octadec-9-enoyl]tetrahydropyridazine-3,6-dione (6b)

White powder, yield = 70%, m.p. = 73°C, IR (KBr, cm^-1^): 3229 (N-H), 2922 (C-H asymm), 2854 (C-H symm.), 1738 (C=O), 1689 (CONH, lactam ring). ^1^H-NMR (400MHz, CDCl_3_): δ 8.34 (s, 1H, NH), 5.40 (m, 2H, -C*H*=C*H*-), 2.83-2.60 (m, 4H, -C*H_2_*-C*H_2_*-, ring), 2.36 (t, 2H, J = 7.5 Hz, C*H_2_*-CO), 1.99 (m, 2H, C*H_2_*-CH=CH-C*H_2_*), 1.68 (m, 2H, C*H_2_*-CH_2_-CO), 1.31 (br.s, 20H, chain-C*H_2_*), 0.87 (dist.t, 3H, terminal-C*H_3_*). ^13^C-NMR (CDCl_3_, Proton decoupled): δ 173.2, 170.0 (2*C*=O, ring), 167.3 (*C*=O, chain), 131.4 (C_9_, -*C*H=CH-), 129.8 (C_10_, -CH=*C*H-), 35.6 (C_2_), 32.8, 30.3 (2C, -*C*H_2_-*C*H_2_-, ring), 30.0, 29.8, 29.6, 29.2, 29.1, 28.8, 28.6, 27.2, “one signal hidden“, 26.7, 25.3(C_3_-C_8_, C_11_-C_17_, chain-*C*H_2_), 14.1 (C_18_, *C*H_3_). ESI/MS (*m/z)*: 378.4 (M^+^).

### 1-Hexadecanoyltetrahydropyridazine-3,6-dione (6c)

Yellow powder, yield = 68%, m.p. = 72–74°C, IR (KBr, cm^-1^): 3238 (N-H), 2924 (C-H asymm.), 2857 (C-H symm.), 1734 (C=O), 1678 (CONH, lactam ring). ^1^H-NMR (400MHz, CDCl3): δ 8.06 (s, 1H, NH), 2.73-2.41 (m, 4H, -C*H_2_*-C*H_2_*-, ring), 2.20 (t, 2H, J = 7.3 Hz, C*H_2_*-CO), 1.53 (m, 2H, C*H_2_*-CH_2_-CO), 1.21 (br.s, 28H, chain-C*H_2_*), 0.83 (dist.t, 3H, terminal-C*H_3_*). ^13^C-NMR (CDCl_3_, Proton decoupled): δ 172.8, 170.1 (2*C*=O, ring), 168.6(*C*=O, chain), 34.3 (C_2_), 33.1, 32.2 (2C, -*C*H_2_-*C*H_2_-, ring), 30.1, 29.9, 29.7, 29.6, 29.4, 29.1, 28.8, 28.7, 28.3, 27.2, 24.9, 24.6, 23.1 (C_3_-C_15_, chain-*C*H_2_), 14.0 (C_16_, *C*H_3_). ESI/MS (*m/z)*: 352.4 (M^+^).

## Conclusion

A series of compounds, **3a–d** and **6a–c**, were synthesized from fatty acid hydrazides, CDI, and succinyl chloride. The compounds were evaluated for their *in vitro* cytotoxicity on the MDA-MB-231, KCL-22, and HeLa cell lines using an MTT-based assay. The IC_50_ values and dose-dependent graphs revealed that nearly all the synthesized compounds exhibited good or moderate anticancer activity at their specific concentration range. The results showed that compounds **3b**, **3c**, and **6b** displayed potent activity against the specific cell line. The structure-activity relationship study showed that the nature of the fatty acid chains strongly determines the anticancer potency of compounds. It was found that 1,3,4-oxadiazol-2(3*H*)-one and tetrahydropyridazine-3,6-dione moieties with an unsaturated fatty acid have more anticancer activity than the saturated analogues towards the three tested cell lines. In conclusion, our findings might be beneficial for designing new 5-membered or 6-membered heterocyclic moieties with embedded fatty acid chains as potential anticancer drugs for therapeutic use.
